# Sterilization of the Unfit

**Published:** 1910-12

**Authors:** G. Henri Bogart

**Affiliations:** Brookville, Indiana


THE
### TEXAS MEDICAL JOURNAL.
Established July, 1885

F. E. DANIEL, M. D., - - - - - - Editor, Publisher and Proprietor

Published Monthly.—Subscription $1.00 a Year.

VOL. XXVI AUSTIN, DECEMBER, 1910. No. 6

The publisher is not responsible for the views of the contributors.

### Original Articles.

For Texas Medical Journal.

### Sterilization of the Unfit.

BY G. HENRI BOGART, M. D., BROOKVILLE, INDIANA.

Ever since my paper on the "Indiana Plan," of sterilizing the unfit appeared in the September number of the TEXAS MEDICAL JOURNAL letters have been pouring in from physicians of that State.

One gentleman tells me of an obstetric case, third child, in which the mother had an attack of epilepsy during labor, both other children epileptics; the latter a condition almost certain.

Which were better, to allow these burdens of suffering to become a plague upon themselves, or to allow them to remain unborn?

Quite a number have inquired whether the retention of orchitic fluid and spermatozoa might not lead to increased sexual desire and power, and hence make the subjects dangerous to society. The answer, empirically, is "no." The something over 600 cases from our reformatory afforded exceptional opportunities for record, as after release, our prisoners are on parole for definite periods, with compulsory reports, and not one instance has occurred of either loss of, or excess of copulative power.

Theoretically, too, the reflex impetus to sexual desire follows the filling of the seminal vesicles, and this will follow without its impregnation with spermatozoa. Semen is ejaculated, after vasectomy as before.

Remember it as an axiom of sterilization; power and desire remain practically unchanged in the male; and are only slightly increased in the female.

Very many have asked why rapists were included. Our law was passed by the narrowest margin of majority, and on the last day of the session, and it was difficult enough to get the votes from those who did not understand the matter. And when they insisted on including the rape fiend, our folks had to acquiesce. However, the direction in our law is: "Such operation as shall be decided as safest, and most effective," remained.

Under this reading I can assure you that rapists may be and are given adequate care.

There was an error crept into the Connecticut law, to which I failed to call attention in my previous paper—the use of the word "oophorectomy" instead of "Fallopian Section."

Dr. Harry C. Sharp, the father of vasectomy as a preventive of criminal reproduction, is still connected with the Indiana reformatory, as trustee, and may be reached by mail at West Baden Springs, Indiana. I do not wish the readers of these papers to accept my word alone, and the Doctor will corroborate what I may say, I am sure.

When the International Prison Congress was on a tour of inspection of American prisons last autumn, Dr. Sharp demonstrated the operation to the delegates, only to receive their universal approbation, and last week I received an official letter from the national offices of the Florence Crittenton Mission, indorsing the paper in the "Red Back" (TEXAS MEDICAL JOURNAL, Austin, Texas).

The press has devoted much space recently to the fact that judicious selection of seed corn has added more than fifty millions of dollars to the corn crop of Iowa.

Good work and commendable, but if corn be worth the effort, what of men and women?

Is humanity worth as much as corn?

Texas has shown a grasp upon great economic questions, which is the surprise of the American people.

Can she come to the front with a law which shall sterilize at least her institutional unfit, so that they will not pass their unutterable burden of woe and horror into the future?

Allow me to relate the story of "Blind Bill" as an example from my own experience.

After I had held an inquest over his body I investigated his career. He was born illegitimately to a 14-year-old mother, one of a degenerate family. He was a bright, winsome child.

At the age of 3 a gonorrheal inflammation destroyed his eyes. Then when old enough he was sent to the institution for the blind, and made a commendable pupil until puberty, when he became a ferocious masturbator. His habit, too, was particularly disgusting, he thrusting his fingers in cone shape into the anus. When he reached his majority he became so maniacal that he was confined in the poorhouse, to protect himself and others from his violence. He became more and more bestial until the county was compelled to build an isolated cell house, with a removable wooden floor, and one of the inmates specially to watch over him. For years it was impossible to keep clothing of any kind upon him, and the power of coherent speech excepting only the most horrible torrents of obscenity and profanity I ever listened to, passed from him.

Finally, at past 50, he died, and I shipped his body to the State Anatomical Board for dissection.

In Indiana we ship all unclaimed pauper bodies to a State board, which furnishes subjects to all duly authorized schools and individuals at actual cost. This man was a charge for outside poor relief as a baby, and an expense upon civilization his entire life, the only service that he rendered in the end being the little afforded at the dissecting table.

Of the family, none has reached up, out of the plane of degradation, from which they sprang.

The only distinction is when some of them graduate into the poorhouse, or the penitentiary, and in the latter exigency their future procreation stops.

Review such a career, dear Doctor,—review the careers of families to be found in every community. Balance the good and ill of these cases, not only to themselves individually, but to the vaster, more important element of human development.

The photo was made for me by John W. Baker. Study it. Go back, Doctor, re-read my paper in the September TEXAS MEDICAL JOURNAL. Then be up and doing.

Your legislator will have this question to decide at the coming session. You can furnish him the necessary scientific knowledge. When the law was up in our Legislature for 1907, Dr. Horace Read was a member. At the commencement of the session, Dr. Read consulted with the State Boards of Charities and of Health, and decided to devote his entire energies to the passage of the anti-procreation law, and he succeeded, by the narrowest of margins. Some man must put himself behind any great movement ere it crystallizes into fact. The movement will not require so great effort that it has been demonstrated. The evils that were anticipated have proven unfounded, the complications that were feared have faded like mist before the morning sun.

I know of no element of human progress that has so nearly met all the expectations for good, and has remained so entirely free from all evils anticipated or otherwise, as has sterilization by vasectomy.

Dr. Sharp first began operating on criminals in the reformatory in September, 1899, and read a paper describing forty cases, in October of the same year, before the Mississippi Valley Medical Association, at Put-in-Bay. After that he remained silent, devoting his energies to studying the phases of the operation, the results and effects and to securing the enactment of the measure into law. All that he hopes for, all that I hope for, is the extension of this wonderful boon. I make this statement because there are certain people who are challenging Dr. Sharp's title as the father of sterilization, and with its international development there will be more and more attempts to gain cheap notoriety from it.

Vasectomy or vas occlusion is old, old as human history. In the female, as a criminal operation, it has been universally known among the unprincipled surgeons for many years.

The world recognizes accomplishment. Daguerre did not make the first sun picture, nor was Fulton the first to build a boat propelled by steam, yet they were the ones who brought about the crystallization of success, and their names shall link permanently with their discoveries, even as Sharp's shall with vasectomy.

Sterilization is only commencing. The possibilities are wide. Reader, shall Texas be the first of the Southern States to join this phase of progress? The success of the measure lies largely with the physicians of that State.

